# Aptasensor for multiplex detection of antibiotics based on FRET strategy combined with aptamer/graphene oxide complex

**DOI:** 10.1038/s41598-019-44051-3

**Published:** 2019-05-21

**Authors:** Hyungjun Youn, Kwanghyun Lee, Jin Her, Jinseong Jeon, Jihyun Mok, Jae-in So, Sangeon Shin, Changill Ban

**Affiliations:** 1 Department of Chemistry, Pohang University of Science and Technology, 77, Cheongam-Ro, Nam-Gu, Pohang, Gyeongbuk 37673 South Korea; 2 Department of Life Sciences, Pohang University of Science and Technology, 77, Cheongam-Ro, Nam-Gu, Pohang, Gyeongbuk 37673 South Korea; 3 Department of Interdisciplinary Bioscience and Bioengineering, Pohang University of Science and Technology, 77, Cheongam-Ro, Nam-Gu, Pohang, Gyeongbuk 37673 South Korea

**Keywords:** DNA, DNA

## Abstract

The development of a multiplexed sensing platform is necessary for highly selective, sensitive, and rapid screening of specific antibiotics. In this study, we designed a novel multiplex aptasensor for antibiotics by fluorescence resonance energy transfer (FRET) strategy using DNase I-assisted cyclic enzymatic signal amplification (CESA) method combined with aptamer/graphene oxide complex. The aptamers specific for sulfadimethoxine, kanamycin, and ampicillin were conjugated with Cyanine 3 (Cy3), 6-Carboxyfluorescein (FAM), and Cyanine 5 (Cy5), respectively, and graphene oxide (GO) was adopted to quench the fluorescence of the three different fluorophores with the efficiencies of 94.36%, 93.94%, and 96.97% for Cy3, FAM, and Cy5, respectively. CESA method was used for sensitive detection, resulting in a 2.1-fold increased signal compared to those of unamplified method. The aptasensor rapidly detected antibiotics in solution with limit of detection of 1.997, 2.664, and 2.337 ng/mL for sulfadimethoxine, kanamycin, and ampicillin, respectively. In addition, antibiotics dissolved in milk were efficiently detected with similar sensitivities. Multiplexed detection test proved that the fluorescently modified aptamers could work separately from each other. The results indicate that the aptasensor offers high specificity for each antibiotic and enables simultaneous and multicolor sensing for rapid screening of multiple antibiotics at the same time.

## Introduction

Antibiotics, which are commonly used in animal husbandry and agriculture, play a major role in the food industry^1^. However, overusing antibiotics such as sulfadimethoxine, kanamycin, and ampicillin may leave undesirable residues in food products^2–4^. These can cause serious side effects, such as allergic reactions, chemical poisoning, vomiting, and diarrhea^5^. Therefore, maximum residue limit (MRL) has been defined by the Food and Drug Administration, Food and Agriculture Organization of the United Nations/World Health Organization, and Korea Food and Drug Administration to monitor extremely high concentrations of antibiotics in food^6–8^. To detect residual antibiotics in food products, a variety of methods such as high-performance liquid chromatography (HPLC), capillary electrophoresis (CE), and enzyme-linked immunosorbent assay (ELISA) are conducted^9–11^. HPLC is the most widely used method and shows high selectivity and sensitivity; however, this method requires expensive sample preparation and equipment^12^. CE also requires expensive instrumentation and ELISA is limited in practice because of its reliance on the surrounding environment^13,14^. Thus, inexpensive, rapid, accurate, and sensitive methods for detecting residual antibiotics in food must be developed.

Aptamers are single-stranded DNA oligomers that can be used as probes for proteins, cells, and small molecules and bind to target molecules with high affinity^15–17^. Various types of aptamer-based sensors have been developed to improve the traditional antibody-based assays because of their advantages over antibodies such as low cost, easy synthesis, and high chemical stability^18–22^. Furthermore, aptamer has great advantages for small molecule detection over the antibody. Molecules which are smaller than molecular weight 2000 g/mol, usually have not enough immunogenicity to generate specific antibody^23^. Therefore, the polypeptide moiety is necessary as a carrier to make immune response with small molecules, which is called hapten. This moiety could have binding affinity to the developed antibody, thus the antibody should have low specificity for the target small molecule. However, the small molecule aptamers show great affinity and specificity to the target small molecule over the other biomolecules, based on its discovery process called systematic evolution of ligands by exponential enrichment (SELEX). In SELEX process, the target is directly bound to aptamer libraries without any other carrier proteins, by immobilization of the targets on magnetic beads or agarose^24^. Consequently, the aptamer-based sensors were found to be inexpensive, selective, and sensitive.

Graphene oxide (GO), which has multiple oxygen-containing groups on its surface, is widely used in the field of sensing because of its unique characteristics, such as facile surface modification, large surface area, strong photoluminescence, and good water dispersibility^25–29^. Furthermore, because of its non-radioactive electronic excitation energy transfer and large absorption cross-sections^30^, GO can be used to develop fluorescence resonance energy transfer (FRET) sensors^31–34^. A sensing platform was developed according to the noncovalent binding of a fluorophore-labeled aptamer to GO, which was induced by π-π stacking^35,36^, resulting in FRET from the fluorophore to GO. FRET resulted in highly efficient quenching of the fluorophore. Addition of antibiotics caused the dissociation of the fluorophore-labeled aptamer from GO, recovering the fluorescence intensity.

Herein, an inexpensive, rapid, selective, sensitive, and water-soluble fluorescence aptasensor was developed using GO as a quenching material. The fluorescence modifiers such as Cyanine 3 (Cy3), 6-Carboxyfluorescein (FAM), Cyanine 5 (Cy5) were selected to develop the aptasensor since the fluorescence-based target detection have advantages over multiplexed sensing schemes^37^. A sulfadimethoxine-specific aptamer labeled with Cy3, a kanamycin-specific aptamer labeled with FAM, and ampicillin-specific aptamer labeled with Cy5 were used as antibiotic binding probes, which have different emission and excitation wavelengths^38^. In the absence of antibiotics, aptamers labeled with fluorophores adsorbed on the GO surface, inducing FRET between the fluorophore and GO. Subsequently, the fluorescence emissions were rapidly quenched. However, in the presence of antibiotics, each antibiotic interacted with its corresponding aptamer labeled with the fluorophore and switched the conformation of the aptamers, preventing the adsorption of GO. This recovered the fluorescence intensity in the sensing platform. In this study, we developed an aptasensor for antibiotics based on target-induced conformational changes of aptamers modified by fluorophores and subsequent changes in their interactions with GO.

## Results and Discussion

### Principle of CESA-based fluorescent aptasensor for antibiotics

**Figure 1:**
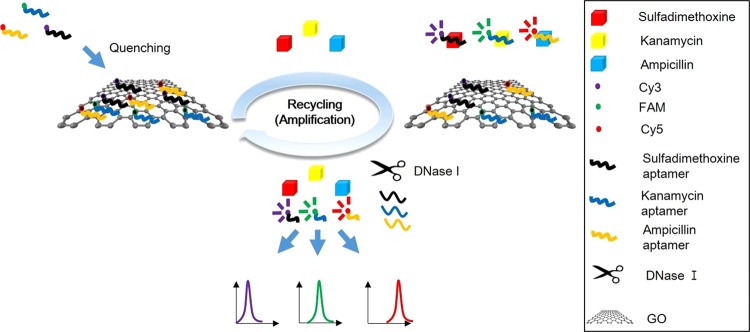
Schematic illustration of a high-efficient multiplex GO-based aptasensor for the detection of antibiotics using CESA.

A FRET-based aptasensor for antibiotics was developed using antibiotic-induced fluorescence signal changes by recording the spectrum of each channel such as Cy3, FAM, and Cy5. In the presence of each antibiotic, the aptamer bound to the antibiotic via strong affinity effects, such as hydrogen bonding, electrostatic forces and spatial matching, prevented the adsorption of GO. Then, the unbound fluorophore-labeled aptamer exhibited a fluorescence signal according to the concentration of antibiotics. In this step, to increase the overall fluorescence intensity, the cyclic enzymatic signal amplification (CESA) method is applied to the aptasensor by addition of DNase I for sensitive detection by recycling the antibiotics. The cleaved fragments of aptamer labeled with fluorophores still emitted fluorescence; thus, FRET did not occur and a clear fluorescence signal was detected. To increase detection accuracy, aptamers adsorbed on GO were removed by centrifugation. The overall scheme is shown in Fig. 1.

In contrast, in the absence of target antibiotics, a fluorophore-labeled aptamer bound GO via electrostatic interactions and minor π-π stacking^39^, quenched the fluorescence signal by FRET (Fig. 1). Using this strategy, quantitative results for each antibiotic were obtained using a CESA-based fluorescent aptasensor.

### Optimization of detection conditions

The designed aptasensor was tested to determine the optimal sensing conditions for antibiotics using the CESA method. Based on the principle of the proposed biosensor, antibiotic detection was achieved by binding between fluorophore-labeled aptamers and GO. First, the concentration of GO was optimized. Various concentrations of GO (0.5, 1, 1.5, 2, 3, 4, and 5 μg/mL) were added to 20 mM Tris-HCl buffer (5 mM MgCl_2_ and 15 mM NaCl, pH 8.0) at fixed concentrations of 100 nM sulfadimethoxine aptamer (P1), 100 nM kanamycin aptamer (P2), and 100 nM ampicillin aptamer (P3). When more than 3 μg/mL of GO was added to the solution, the fluorescence intensities of Cy3, FAM, and Cy5 were reduced to nearly zero and were not recovered by the addition of sulfadimethoxine (T1), kanamycin (T2), and ampicillin (T3) (Supplementary Fig. S1). Therefore, 2 μg/mL of GO was selected to detect P1, P2, and P3 because it showed high quenching efficiency for P1, P2, and P3. Additionally, in the presence of T1, T2, and T3, the sample showed a detectable intensity change. Therefore, in subsequent experiment, the GO concentration was fixed at 2 μg/mL.

**Figure 2:**
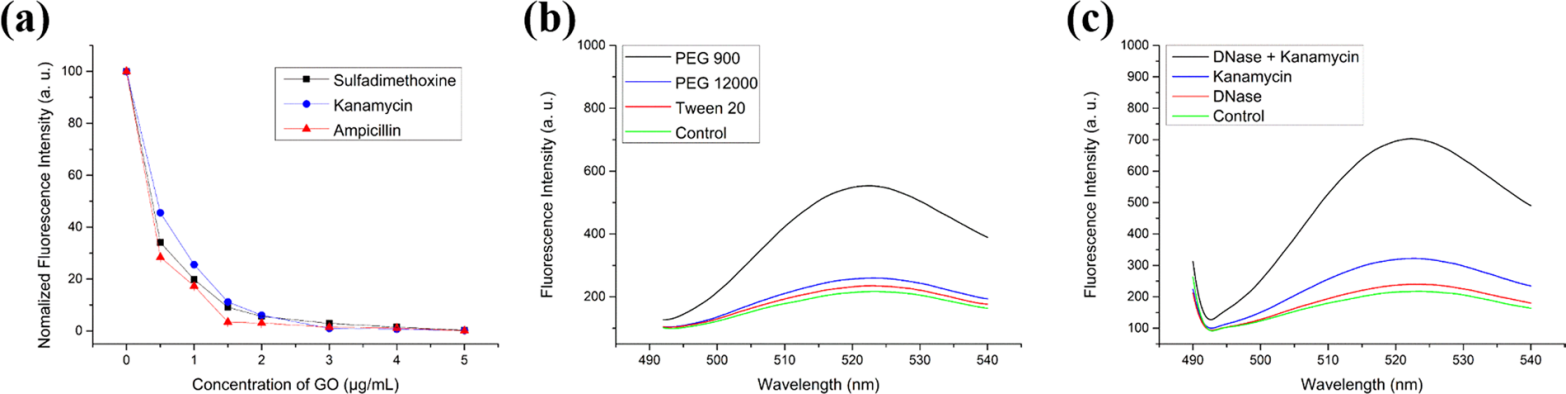
(**a**) Graphene oxide quenching efficiency (**b**) Blocking agent efficiency (**c**) DNase I effect.

A surface-blocking agent is commonly used to enhance the efficiency of a biosensor^40^. However, the surface of GO is unique because of its heterogeneity^41,42^. Therefore, blocking the GO surface may require more sophisticated control of surface forces. If a blocking agent is considerably weak, a blocking effect cannot be achieved. In contrast, if a blocking agent is markedly strong, probe adsorption may be affected, resulting in a weak signal. In this study, blocking agents (polyethylene glycol (PEG) MW 900, PEG MW 12000, and Tween 20) were compared for their abilities to sense antibiotics^43^. Under optimized conditions, the sensitivities to target antibiotics were improved. Considering the principle of the sensing system, adequate binding affinity was achieved between the fluorophore-labeled aptamers (P1, P2, and P3) and GO. If fluorophore-labeled aptamers (P1, P2, and P3) have stronger affinity for GO than for the antibiotics (T1, T2, and T3), it would be considerably difficult to produce a fluorescence signal. Also, the antibiotics would directly bind to GO which results significant reduction of the interaction between the antibiotics and its aptamers. Therefore, one method for improving the analytical performance of the aptasensor is to block the GO surface using a blocking agent to avoid those various non-specific interactions.

In this study, PEG MW 900, PEG MW 12000, and Tween 20 were screened to compare the efficiency of fluorescence recovery. Buffer A (20 mM Tris-HCl, 5 mM MgCl_2_, 15 mM NaCl, and PEG MW 900 0.001%, pH 8.0), buffer B (20 mM Tris-HCl, 5 mM MgCl_2_, 15 mM NaCl, and PEG MW 12000 0.001%, pH 8.0), and buffer C (20 mM Tris-HCl, 5 mM MgCl_2_, 15 mM NaCl, and Tween 20 0.001%, pH 8.0) were used. In the experiments, 2 μg/mL of GO was added to buffer A, buffer B, and buffer C, while 10 ng/mL T2 was added to 100 nM of P1, P2, and P3 for 30 min at room temperature. Subsequently, solutions of T2, P1, P2, and P3 were added to the buffer including each blocking agent for 30 min at room temperature. These samples were centrifuged for 15 min at 4000 g at 4 °C and analyzed with a fluorometer. The blocking agent effects on enhancement of fluorescence signal were compared by fluorescence recovery rate which is defined as the ratio fluorescence intensity in each buffer over fluorescence intensity of control. The fluorescence recovery rate of buffer A was 2.1-fold higher than that of buffer B and 2.4-fold higher than that of buffer C (Fig. 2b). Thus, buffer A was selected for subsequent experiments.

### Cyclic enzymatic signal amplification with DNase I

To improve analytical performance of the GO-based FRET strategy, CESA using DNase I was conducted in the sensing system^44,45^. GO has been reported to exhibit the protective property against DNase I, which is a non-specific endonuclease that cleaves single-stranded DNA. Upon the addition of specific antibiotics, the fluorescent-labeled aptamer bound to the antibiotics and the aptamer-antibiotic complexes desorbed from GO, with the fluorescent-labeled aptamer emitting fluorescence. After the addition of DNase I, the aptamers were digested, inducing recycling of the antibiotics. The recycled antibiotics then released another fluorophore-labeled aptamer from GO, initiating the next round of cleavage and resulting in fluorescence signal amplification. Therefore, this platform utilizing DNase I achieved high sensitive analytical performance.

First, the protective property of GO against DNase I was examined. Mixture of three aptamers with total concentration of 100 nM was used to this test at 2 μg/mL of GO in buffer A as mentioned above. The aptamers were visualized with 3% (w/v) agarose gel electrophoresis (Supplementary Fig. S2). Addition of GO into the mixture of the aptamers abandoned the aptamer band in gel electrophoresis, suggesting that there are no free aptamers in the buffer with 2 μg/mL of GO. To detach the aptamers from GO, the samples were boiled, resulting recovery of the aptamer band. Likewise, addition of DNase I also abandoned the aptamer band from gel electrophoresis. However, a boiled sample after the addition of GO and DNase I in a row, showed the intact aptamer band. These results support that GO have strong protective property for the single-stranded aptamers against DNase I, while the aptamers are stuck in the GO surface.

Further, to evaluate the effects of DNase I, the response to 10 ng/mL T2 was analyzed in the absence and presence of DNase I. GO (2 μg/mL) was added to 20 mM Tris-HCl buffer (5 mM MgCl_2_, 15 mM NaCl, and 0.001% PEG MW 900, pH 8.0) while 10 ng/mL T2 was added to 100 nM of P1, P2, and P3 for 30 min at room temperature. Next, 10 U of DNase I were added and incubated for 30 min at 37 °C. The samples were centrifuged for 15 min at 4000 g at 4 °C. The fluorescence intensity was analyzed using the FAM channel (excitation at 480 nm and emission at 520 nm). Only the sample treated with DNase I showed a slight increase compared to the control, indicating that the fluorescence signal from only DNase I is negligible. Additionally, the sample treated with DNase I in the presence of T2 showed a 2.1-fold increase in fluorescence recovery rate compared to the untreated sample (Fig. 2c). Because of this increase in fluorescence intensity, a value of 10 U of DNase I was used for subsequent experiments.

### Detection of antibiotics using the CESA strategy

**Figure 3:**
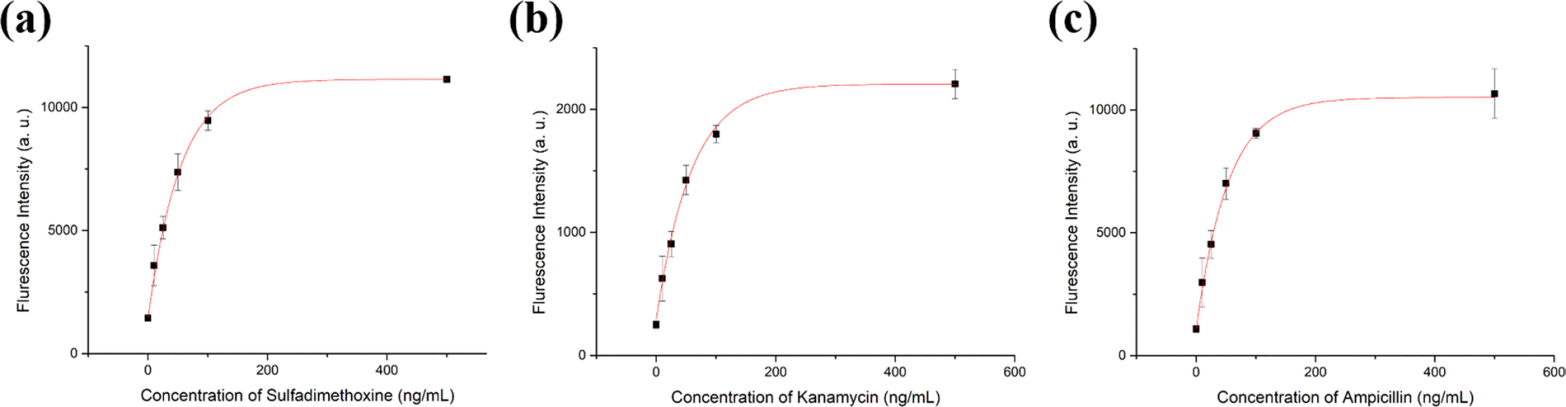
(**a**) The quantification curve from fluorescent detection of sulfadimethoxine in buffer. (**b**) The quantification curve from fluorescent detection of kanamycin in buffer. (**c**) The quantification curve from fluorescent detection of ampicillin in buffer. The curve represents the fluorescent intensity versus concentration of antibiotics. The increments of fluorescent intensity were measured from 10 ng/mL to 500 ng/mL. Each fluorescent intensity was measured 3 times under same conditions. Standard deviations are indicated by error bars. Fluorescence intensity was analyzed on each Cy3 channel (excitation at 520 nm and emission at 565 nm), FAM channel (excitation at 480 nm and emission at 520 nm), and Cy5 channel (excitation at 650 nm and emission at 670 nm).

A quantification test of the designed aptasensor was performed using different concentrations of T1, T2, and T3. As shown in Fig. 3, fluorescence recovery was clearly detected compared with the blank sample. The resulting calibration curves for T1, T2, and T3 were obtained from the fluorescence intensity. Various concentrations of T1, T2, and T3 (10, 25, 50, 100, and 500 ng/mL) interacted with P1, P2, and P3, respectively. After incubation with GO in DNase I-treated buffer containing blocking agent, fluorescence intensity was measured using different wavelength channels (Supplementary Fig. S3). As expected, the increase in fluorescence intensity was proportional to the concentration of antibiotics. This aptasensor had a wide dynamic range as shown in Fig. 3. The limit of detection (LOD) was calculated using the 3-sigma method in the range of 10–50 ng/mL. LOD values of 1.997, 2.664, and 2.337 ng/mL for T1, T2, and T3, respectively, were calculated (Table 1).

**Table 1:** LOD and R-square values of antibiotics.

Antibiotics	LOD (Buffer)	LOD (Milk)	MRL (Milk)
sulfadimethoxine	1.997 ng/mL	6.562 ng/mL	Approx. 100 ng/mL
kanamycin	2.664 ng/mL	6.179 ng/mL	Approx. 150 ng/mL
ampicillin	2.337 ng/mL	6.616 ng/mL	Approx. 10 ng/mL

### Demonstration of aptasensor selectivity

Selectivity is another essential factor for a target-specific aptasensor. Although the selectivity of aptamers was verified in a previous study, the selectivity of this CESA-based aptasensor required further analysis. Thus, in this study, a variety of antibiotics such as chloramphenicol, penicillin, carbenicillin, and amoxicillin was applied to the aptasensor at the same concentrations.

**Figure 4:**
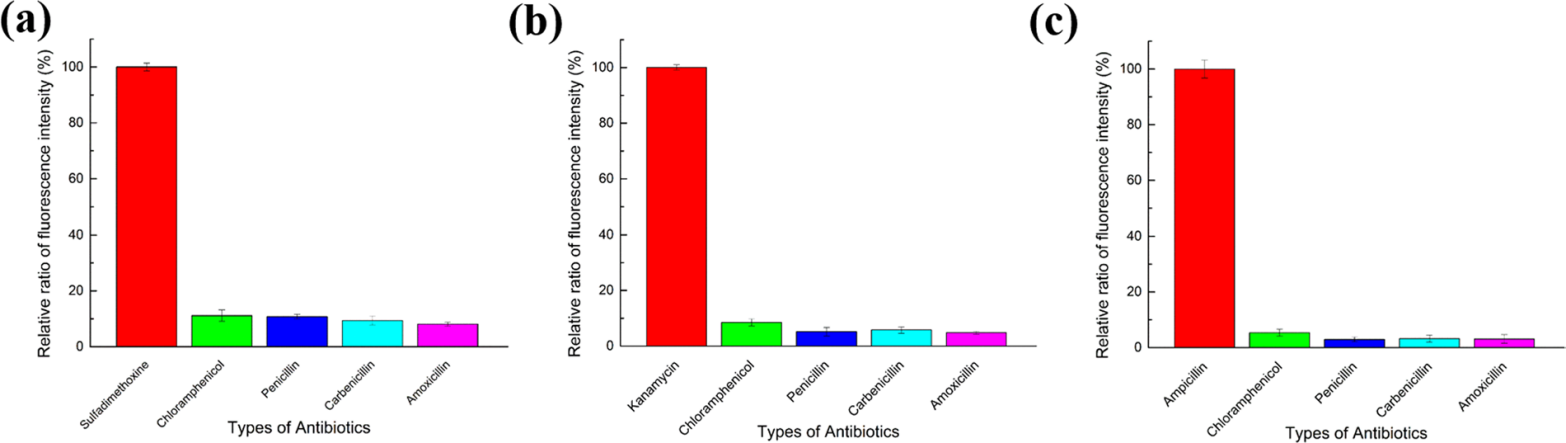
Selectivity test of the aptasensor. The fluorescent signals from several antibiotics are presented relative to the (**a**) sulfadimethoxine, (**b**) kanamycin, and (**c**) ampicillin as the standard. The concentration of all antibiotics were adjusted to 50 ng/mL. Each fluorescent intensity was measured 3 times under same conditions. Standard deviations are indicated by error bars.

GO (2 μg/mL) was added to 20 mM Tris-HCl buffer (5 mM MgCl_2_, 15 mM NaCl, and 0.001% PEG MW 900, pH 8.0), while 50 ng/mL T1 was added to 100 nM of P1, P2, and P3 for 30 min incubation at room temperature. Other antibiotics were also applied to this system under the same conditions as those used for T1. Subsequently, 10 U of DNase I was added and incubated for 30 min at 37 °C. The samples were centrifuged for 15 min at 4000 g at 4 °C. Experiments with T2 and T3 were performed in the same manner. Based on the fluorescence intensities of T1, T2, and T3, the ratios of fluorescence intensities are shown in Fig. 4. The results indicated that the developed aptasensor had good selectivity compared to that of the target signal.

### Detection of antibiotics in milk samples

**Figure 5:**
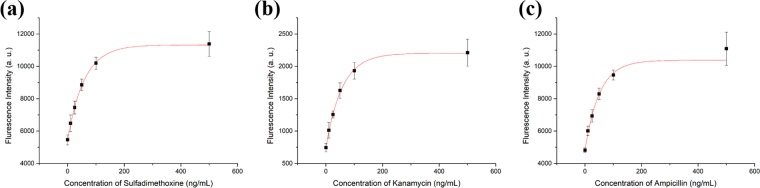
Quantitative detection of spiked antibiotics in milk sample. Shown are the fluorescence intensity measured from 10 ng/mL to 500 ng/mL. Each fluorescent intensity was measured 3 times under the same conditions. Standard deviations are indicated by error bars.

To verify the practicality of the designed CESA-based aptasensor, milk treated with different concentrations of antibiotics were used as a sample. First, the milk sample was pretreated by extraction using ethyl acetate. Next, different concentrations of T1, T2, and T3 (0, 10, 25, 50, 100, and 500 ng/mL) were spiked into the prepared milk. The procedures were the same as those described in Section 3.3. The fluorescence intensity was analyzed for each channel (Fig. 5). Similar to the result of detection in buffer, The LOD calculated using the 3-sigma method in the range of 10–50 ng/mL were 6.562, 6.179, and 6.616 ng/mL for T1, T2, and T3, respectively (Table 1). Additionally, in the milk samples, the aptasensor successfully detected antibiotics with low LOD values that were lower than the respective MRL values for sulfadimethoxine, kanamycin and ampicillin in milk^46–48^.

### Multiplexed detection of antibiotics

**Figure 6:**
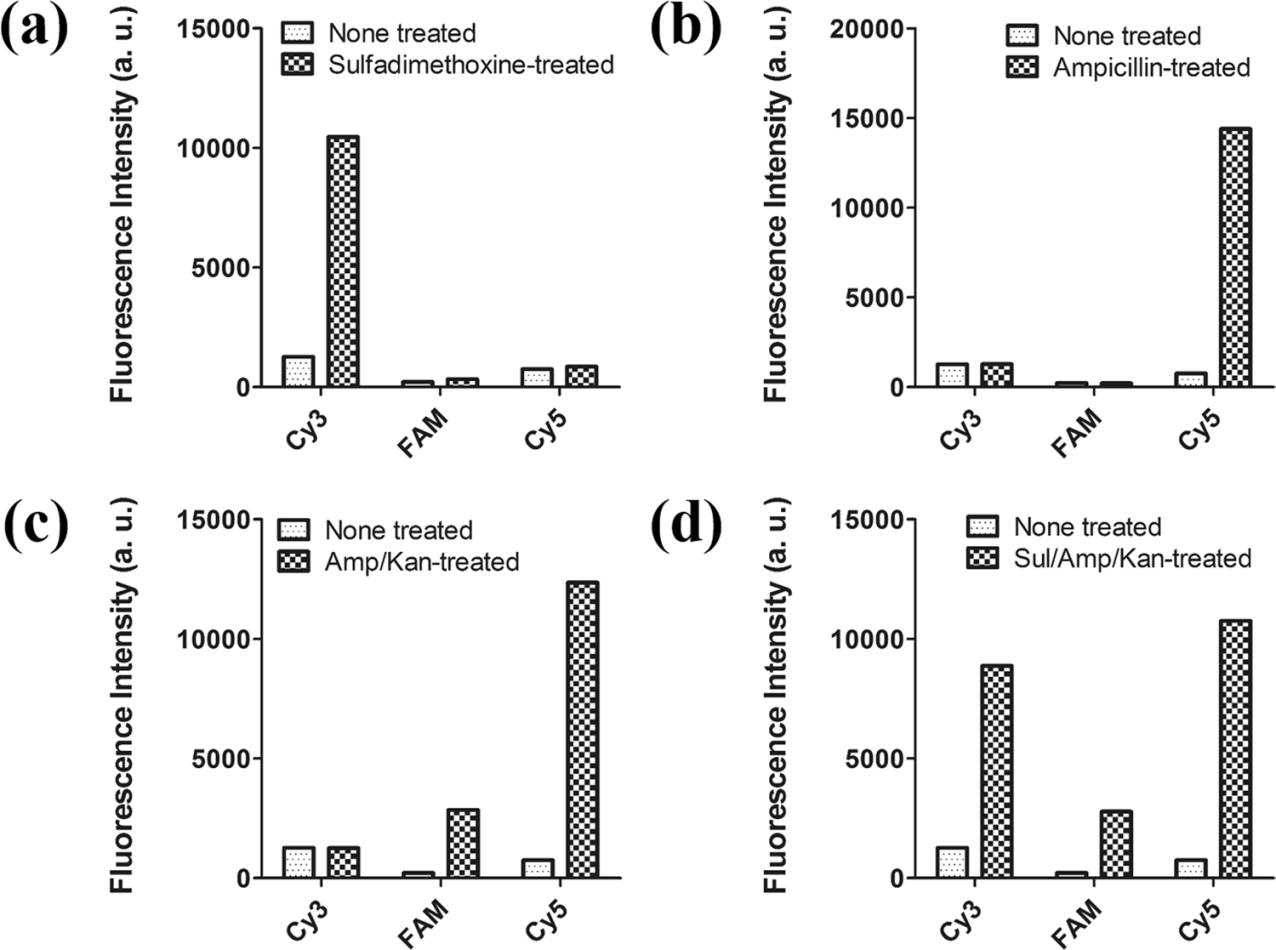
Multiplexed detection of antibiotics. (**a**) 500 ng/mL of sulfadimethoxine was added. (**b**) 500 ng/mL of ampicillin was added. (**c**) 500 ng/mL of kanamycin and 500 ng/mL of ampicillin were added. (**d**) 500 ng/mL of sulfadimethoxine, kanamycin, and ampicillin were all added. Fluorescence intensity was analyzed on each Cy3 channel (excitation at 520 nm and emission at 565 nm), FAM channel (excitation at 480 nm and emission at 520 nm), and Cy5 channel (excitation at 650 nm and emission at 670 nm).

Finally, multiplexed detection of antibiotics was performed. Two or three types of antibiotics were added to the CESA-based aptasensor to validate its multiplexed detection ability. The binding conditions and aptamer concentrations were the same as those described in Section 3.3. For multiplexed detection, dye-to-dye energy transfer was inhibited by using Cy3, FAM, and Cy5, which were excited at 520, 480, and 650 nm, emitting different colors at 565, 520, and 670 nm, respectively. Antibiotic concentrations were fixed at 500 ng/mL. As shown in Fig. 6a, following sulfadimethoxine addition, a fluorescent signal was only observed for the Cy3 channel. Considerably weak signal changes were detected in the FAM and Cy5 channels. Also, the ampicillin channel was tested and showed weak signal changes in the Cy3 and FAM channels (Fig. 6b). Dual detection of kanamycin and ampicillin was also achieved using this system (Fig. 6c). Both kanamycin and ampicillin were added to the system, and signal changes were detected using the FAM and Cy5 channels. In contrast, the Cy3 channel did not show a fluorescence signal. Thus, this method can be used for simultaneous detection of sulfadimethoxine and kanamycin, sulfadimethoxine and ampicillin, and kanamycin and ampicillin. Furthermore, in the presence of all antibiotics, a fluorescence signal was detected in the Cy3, FAM, and Cy5 channels (Fig. 6d). In conclusion, simultaneous detection of two or three antibiotics was successfully performed at a similar level.

## Conclusion

In this study, a highly efficient novel aptasensor was developed for multiplexed detection of antibiotics such as sulfadimethoxine, kanamycin, and ampicillin. Fluorescently modified aptamers, which were quenched by GO adsorption, produced detectable signals once they were removed from GO by competitive binding to corresponding antibiotics. Additionally, the fluorescence intensity was amplified by DNase I. The corresponding peak exhibited a linear relationship with the concentration of each antibiotic. Quantitative detection of antibiotics was successfully implemented with LOD values of 1.997, 2.664, and 2.337 ng/mL for sulfadimethoxine, kanamycin, and ampicillin, respectively, which were calculated in the range of 10–50 ng/mL. In addition, quantitative detection was effectively performed in milk samples. The aptasensor could also successfully detect three antibiotics simultaneously by using the CESA method, which has not been previously reported. The verified aptasensor can be used for simple, rapid and multiplexed detection of antibiotics compared with current assays for antibiotics (Supplementary Table S1) even under MRL values. Furthermore, because of its facile manipulation, simple preparation, and good analytical performance, this method meets the various detection requirements for antibiotics, which are comparable to those of conventional sensing systems.

## Materials and Methods

### Materials and apparatus

Table 2 shows the aptamers used in this study which were synthesized by Cosmogenetech (Korea)^49–51^. The modifications of fluorophores were applied on 5′-end of the aptamers for minimization of interruption to binding affinity of the aptamers based on structural insights from secondary structure predictions (Supplementary Fig. S4). Carbenicillin was obtained from Novagen (Billerica, MA, USA). Kanamycin sulfate and ampicillin sodium salt were purchased from Affymetrix USB products (Santa Clara, CA, USA). Penicillin G sodium salt, amoxicillin trihydrate, chloramphenicol, sulfadimethoxine sodium salt, and Tween 20 were purchased from Sigma-Aldrich (St. Louis, MO, USA). GO was obtained from Cheap Tubes, Inc. (Cambridgeport, VT, USA). PEG MW 900 and PEG MW 12000 were purchased from Fluka Chemika (Buchs, Switzerland). DNase I was purchased from New England Biolabs (Ipswich, MA, USA). Ethyl acetate was purchased from Samchun Chemical (Pyeongtaek, Korea). Other common chemicals, including sodium chloride (NaCl), magnesium chloride (MgCl_2_), and potassium chloride (KCl), were purchased from Sigma-Aldrich.

**Table 2:** Aptamer sequences used in this study.

Probe	Sequence
Cy3-modified sulfadimethoxine aptamer	5′-Cy3-GAG GGC AAC GAG TGT TTA TAG A-3′
FAM-modified kanamycin aptamer	5′-FAM-TGG GGG TTG AGG CTA AGC CGA-3′
Cy5-modified ampicillin aptamer	5′-Cy5- GCG GGC GGT TGT ATA GCG G-3′

Millipore Milli-Q ultrapure water (Billerica, MA, USA) was used throughout the research. Fluorescence emission spectra were recorded with an RF-6000 fluorometer with Xenon-Arc lamp (Shimadzu, Kyoto, Japan) using 1-cm path length disposable cuvettes. The excitation band width was 15 nm and the emission band width was 5 nm. The fluorescence spectra were obtained with a 600 nm/min scan speed and sensitivity was set as an auto mode.

### General procedure for fluorescence sensing of antibiotics

To detect the antibiotics, GO (2 μg/mL) was added to a 20 mM Tris-HCl buffer (5 mM MgCl_2_, 15 mM NaCl, and 0.001% PEG MW 900, pH 8.0) for 30 min at room temperature. Sulfadimethoxine aptamer, kanamycin aptamer, and ampicillin aptamer (100 nM) were added to an antibiotic sample to form an antibiotic aptamer complex. A series of concentrations of antibiotic solution (0, 10, 50, 100, and 500 ng/mL) was added and mixed well. This antibiotic aptamer complex solution was incubated for 30 min at room temperature with mild shaking, and then GO (2 μg/mL) solution was added to this mixture. The solution was mixed well and incubated for 30 min at room temperature with mild shaking. After the addition of 10 U of DNase I, the solution was incubated for 30 min at 37 °C. GO with the residual aptamer was removed by centrifugation for 5 min at 14,000 g. The amount of the remaining fluorescent-labeled aptamer in the supernatant was recorded using a fluorometer.

### Selectivity test of the aptasensor

The applicability of the aptasensor for detecting antibiotics was validated through a selectivity test. Diverse antibiotics such as chloramphenicol, penicillin, carbenicillin, and amoxicillin were added to the system at the same concentration of 50 ng/mL. Each reaction was performed in Tris-HCl buffer for 30 min at room temperature. The sensing procedure was conducted using the same method. Fluorescence data were collected for each channel such as Cy3, FAM, and Cy5.

### Detection of antibiotics in milk samples

The reliability of detection in practical applications was evaluated by recording the recovery rate in actual milk samples. Milk was obtained from a local supermarket. The milk fat layer was removed, and 2 mL of the milk was mixed with 2 mL of distilled water for 15 min. After adding 7 mL of ethyl acetate, the solution was vigorously shaken by vortex mixing for 15 min. The clear supernatant was collected by centrifuging for 15 min at 4000 g at 4 °C. After adding 7 mL of ethyl acetate to the remaining sample, the vortex mixing and centrifugation step was repeated. Ethyl acetate was subsequently removed from the obtained supernatant by gentle nitrogen blow-down at 40 °C. The extracted precipitate was dissolved in 4 mL of distilled water (Song *et al*.^50^). Milk samples were spiked with different concentrations of antibiotics (0, 10, 50, 100, and 500 ng/mL). All procedures were conducted as described above.

### Multiplexed detection of antibiotics by CESA method

First, 100 nM of Cy3-labeled sulfadimethoxine aptamer (P1), 100 nM of FAM-labeled kanamycin aptamer (P2), and 100 nM of Cy5-labeled ampicillin aptamer (P3) were incubated with an antibiotic sample (various concentrations of sulfadimethoxine, kanamycin, and ampicillin) for 30 min at room temperature. Then, samples were added to a GO solution in 20 mM Tris-HCl buffer (5 mM MgCl_2_, 15 mM NaCl, and 0.001% PEG MW 900, pH 8.0) for 30 min at room temperature. Next, 10 U of DNase I were added and incubated for 30 min at 37 °C. The samples were centrifuged for 15 min at 4000 g at 4 °C. The fluorescence intensity was analyzed for each Cy3 channel (excitation at 520 nm and emission at 565 nm), FAM channel (excitation at 480 nm and emission at 520 nm), and Cy5 channel (excitation at 650 nm and emission at 670 nm).

## Supplementary information

Supplementary Information
