# Supplementary material for: Aptasensor for multiplex detection of antibiotics based on FRET strategy combined with aptamer/graphene oxide complex
Source: Sci Rep. 2019 May 21;9:7659. doi: 10.1038/s41598-019-44051-3 (PMC6529438; doi:10.1038/s41598-019-44051-3)
Supplement: Supplementary file 1 — Supplementary Information [file 41598_2019_44051_MOESM1_ESM.pdf]

Supplementary Information

Aptasensor for multiplex detection of antibiotics based on FRET strategy combined with aptamer/graphene oxide complex

Hyungjun Youn[a,§], Kwanghyun Lee[a,§], Jin Her[a], Jinseong Jeon[b], Jihyun Mok[a], Jae-in So[c], Sangeon Shin[a], Changill Ban[a,*]

[a] Department of Chemistry, Pohang University of Science and Technology, 77, Cheongam-Ro, Nam-Gu, Pohang, Gyeongbuk, 37673, South Korea

[b] Department of Life Sciences, Pohang University of Science and Technology, 77, Cheongam-Ro, Nam-Gu, Pohang, Gyeongbuk, 37673, South Korea

[c] Department of Interdisciplinary Bioscience and Bioengineering, Pohang University of Science and Technology, 77, Cheongam-Ro, Nam-Gu, Pohang, Gyeongbuk, 37673, South Korea

E-mail: yhj1005@postech.ac.kr (H. Youn), kwanghyunlee@postech.ac.kr (K. Lee), jinh0902@postech.ac.kr (J. Her), sung0214@postech.ac.kr (J. Jeon), mokjihyun@postech.ac.kr (J. Mok), sojae91@postech.ac.kr (J. So), kcho0204@postech.ac.kr (S. Shin), ciban@postech.ac.kr (C. Ban)

§ These authors are equally contributed to this work.

* Corresponding author:

Tel: +82 54 279 2127; Fax: +82 54 279 5840; E-mail: ciban@postech.ac.kr; Postal address: Department of Chemistry, Pohang University of Science and Technology, 77, Cheongam-Ro, Nam-Gu, Pohang, Gyeongbuk, 790-784, South Korea

Supplementary Figures

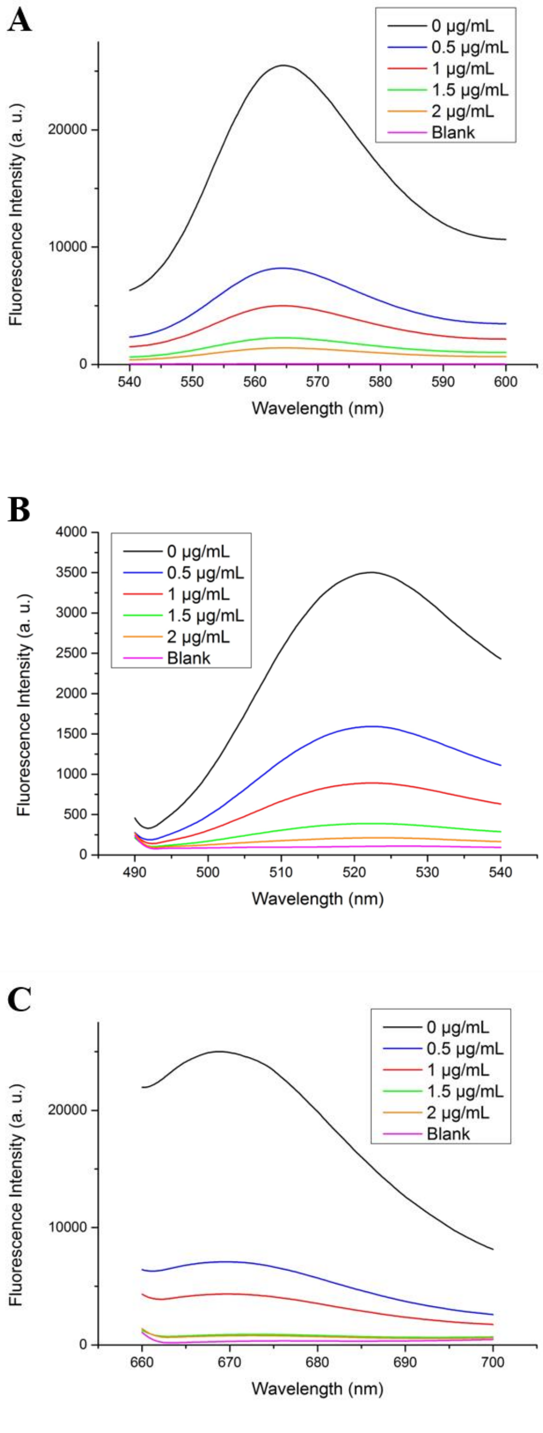

Figure S1. The fluorescence emission spectra of developed system with 100 nM of sulfadimethoxine aptamer (A), kanamycin aptamer (B), ampicillin aptamer (C) in various concentrations of graphene oxide (GO). The spectra showed great quenching efficiency of GO over the fluorophore-labeled aptamers.

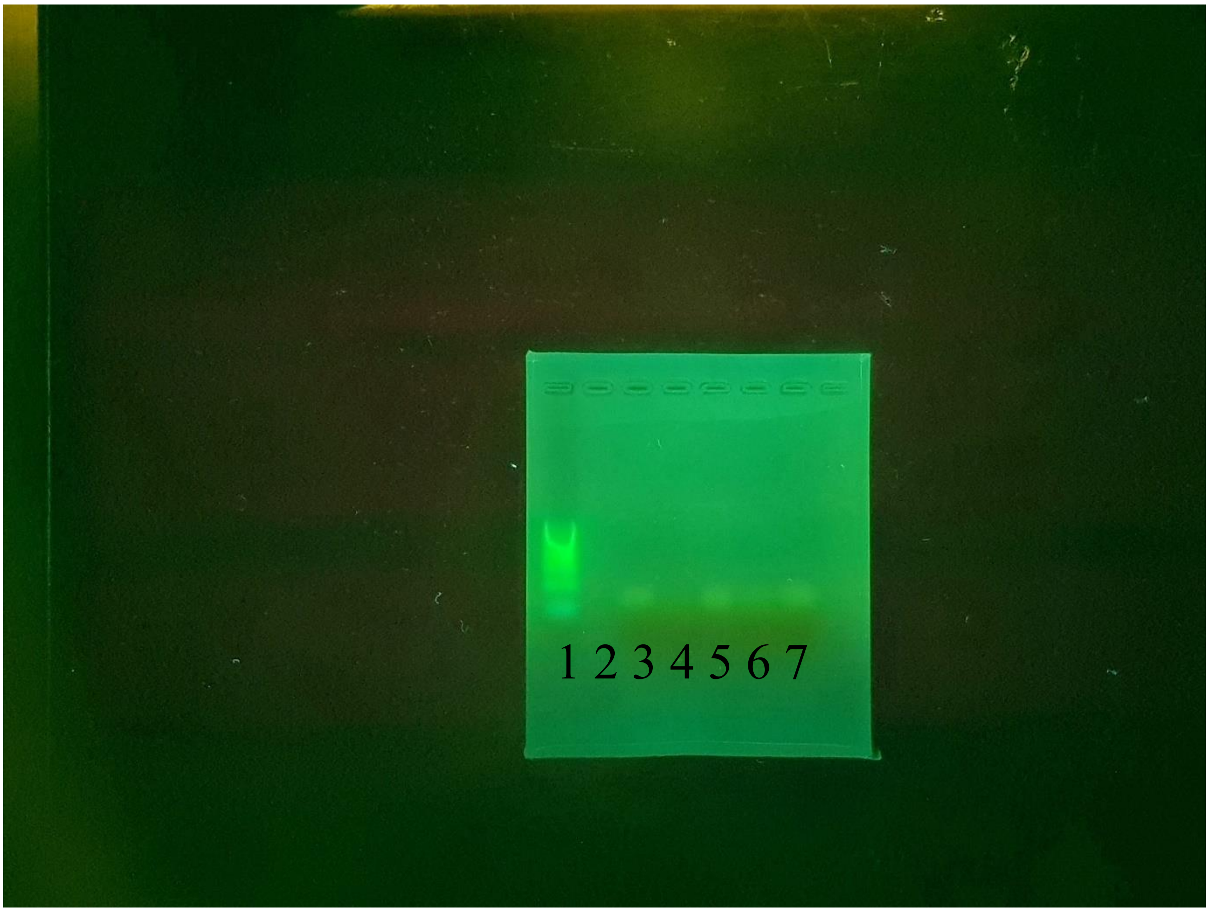
1 2 3 4 5 6 7

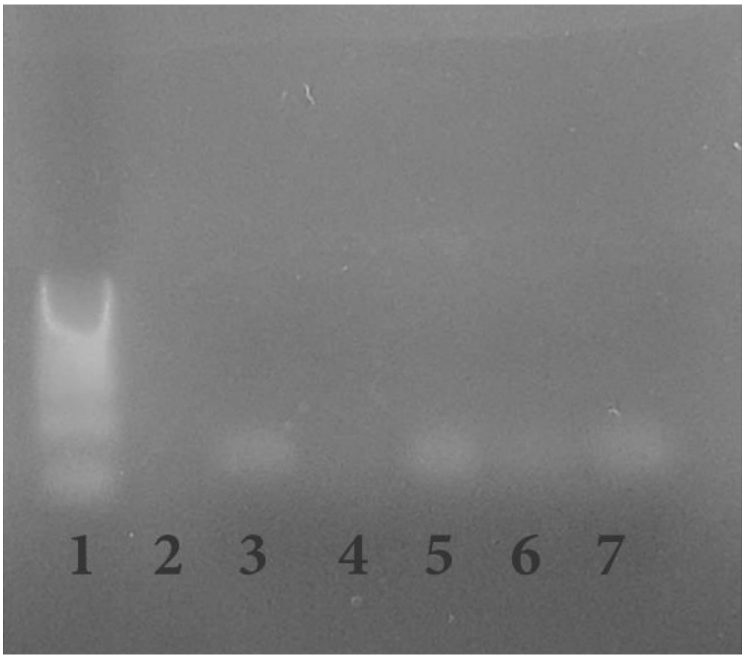
1 2 3 4 5 6 7

Figure S2. DNAse I Activity test with 3 % (w/v) agarose gel electrophoresis

Lane 1: Low range marker (10bp DNA step ladder, Promega, USA)

Lane 2: Mixture of the three Aptamers + GO

Lane 3: Aptamers

Lane 4: Aptamers + DNAse I

Lane 5: Aptamers + GO and Boiling to detach the aptamers from GO

Lane 6: Aptamers + GO + DNAse I

Lane 7: Aptamers + GO + DNAse I and Boiling to detach the aptamers from GO

Addition of GO into the mixture of the aptamers abandoned the aptamer band in gel electrophoresis, suggesting that there are no free aptamers in the buffer with 2 μg/mL of GO (LANE 2). To detach the aptamers from GO, the samples were boiled, resulting recovery of the aptamer band (LANE 5). Likewise, addition of DNase I also abandoned the aptamer band from gel electrophoresis (LANE 4). However, a boiled sample after the addition of GO and DNase I in a row, showed the intact aptamer band (LANE 7). These results support that GO have strong protective property for the single-stranded aptamers against DNase I, while the aptamers are stuck in the GO surface.

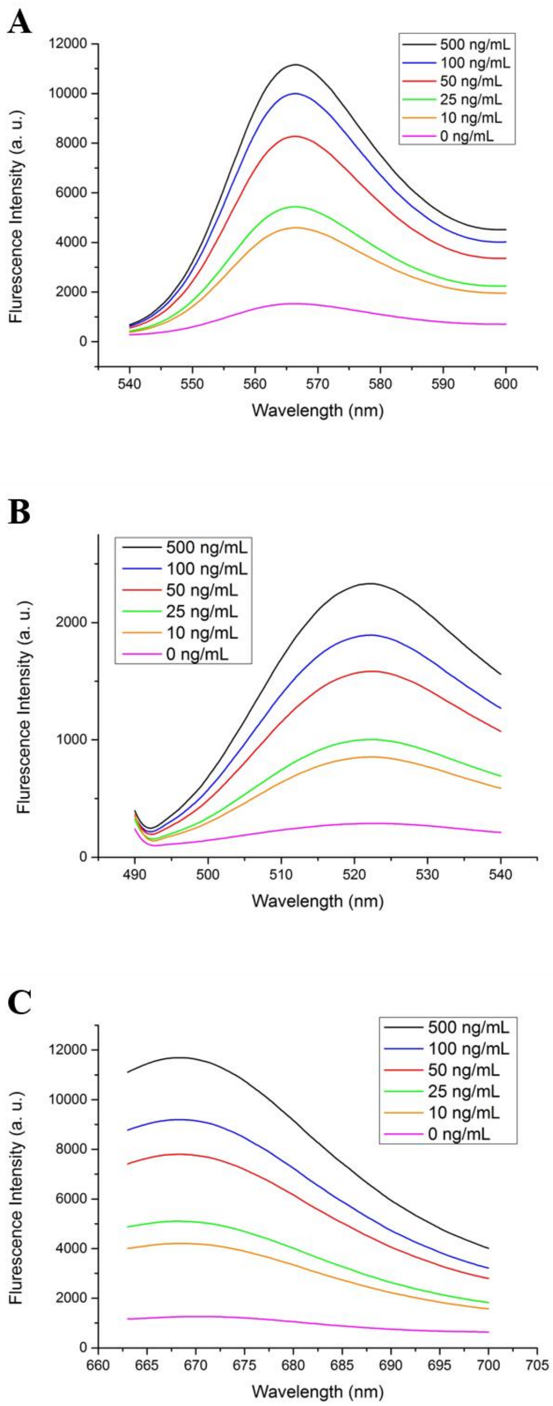

Figure. S3. The fluorescence spectra of developed system with various concentrations of (A) sulfadimethoxine, (B) kanamycin, and (C) ampicillin, respectively. The fluorescence signals were recovered since the modified aptamers were separated from GO.

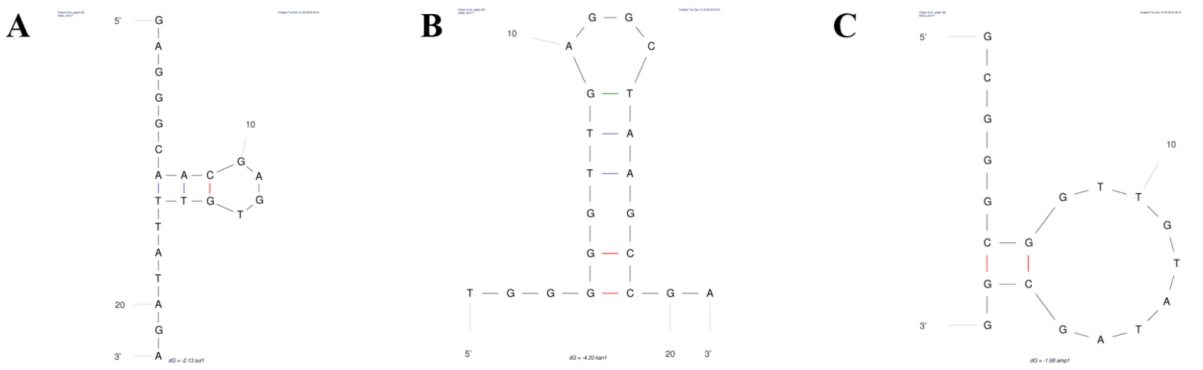

Figure. S4. The predicted secondary structure of (A) sulfadimethoxine aptamer, (B) kanamycin aptamer, and (C) ampicillin aptamer, respectively. The structure predictions were conducted using mfold web server [1].

Target Antibiotics	Detection Scheme	Analysis time	Limit of detection (buffer)	Limit of detection (sample)
Kanamycin (Kan)	Cantilever[2]	10 min	50 uM (24 ug/mL)	N/A
Ampicilin (Amp)	AuNP[3]	1 h	2 ng/mL (fluorescence)<br>10 ng/mL (colorimetry)	2 ng/mL (fluorescence) (milk)<br>10 ng/mL (colorimetry) (milk)
	ELISA[4]	> 3 h	5 ng/mL	5 ng/mL
Sulfadimethoxine (SDM)	AuNP[5]	15 min	50 ng/mL	N/A
	CPNB[6]	> 1 h 35 min	10 ng/mL	10 ng/mL
This study (for all 3 antibiotics stated above)	Multi-fluorescence	> 1 h 30 min for all 3 antibiotics simultaneously	1.997 ng/mL (SDM)<br>2.664 ng/mL (Kan)<br>2.337 ng/mL (Amp)	6.562 ng/mL (SDM)<br>6.179 ng/mL (Kan)<br>6.616 ng/mL (Amp)

Table S1. The comparison between current detection methods for antibiotics and this study. The underlined references are also cited in main manuscript.

References

1 Zuker, M. Mfold web server for nucleic acid folding and hybridization prediction. *Nucleic Acids Research* **31**, 3406-3415, doi:10.1093/nar/gkg595 (2003).

2 Bai, X., Hou, H., Zhang, B. & Tang, J. Label-free detection of kanamycin using aptamer-based cantilever array sensor. *Biosensors and Bioelectronics* **56**, 112-116, doi:10.1016/j.bios.2013.12.068 (2014).

3 Song, K.-M., Jeong, E., Jeon, W., Cho, M. & Ban, C. Aptasensor for ampicillin using gold nanoparticle based dual fluorescence–colorimetric methods. *Analytical and Bioanalytical Chemistry* **402**, 2153-2161, doi:10.1007/s00216-011-5662-3 (2012).

4 Samsonova, Z. V., Shchelokova, O. S., Ivanova, N. L., Rubtsova, Y. M. & Egorov, A. M. Enzyme-Linked Immunosorbent Assay of Ampicillin in Milk. *Applied Biochemistry and Microbiology* **41**, 589, doi:10.1007/s10438-005-0107-4 (2004).

5 Chen, A. *et al.* High sensitive rapid visual detection of sulfadimethoxine by label-freeaptasensor. *Biosensors and Bioelectronics* **42**, 419-425, doi:10.1016/j.bios.2012.10.059 (2013).

6 Song, K.-M., Jeong, E., Jeon, W., Jo, H. & Ban, C. A coordination polymer nanobelt (CPNB)-based aptasensor for sulfadimethoxine. *Biosensors and Bioelectronics* **33**, 113-119, doi:10.1016/j.bios.2011.12.034 (2012).
